# The effects of taurine supplementation on oxidative stress indices and inflammation biomarkers in patients with type 2 diabetes: a randomized, double-blind, placebo-controlled trial

**DOI:** 10.1186/s13098-020-0518-7

**Published:** 2020-01-29

**Authors:** Vahid Maleki, Reza Mahdavi, Fatemeh Hajizadeh-Sharafabad, Mohammad Alizadeh

**Affiliations:** 1 Student Research Committee, Tabriz University of Medical Sciences, Tabriz, Iran; 2 Department of Clinical Nutrition, Faculty of Nutrition and Food Sciences, Tabriz University of Medical Sciences, Tabriz, Iran; 3 Department of Biochemistry and Dietetics, Faculty of Nutrition and Food Sciences, Tabriz University of Medical Sciences, Tabriz, Iran; 4 Nutrition Research Center, Faculty of Nutrition and Food Sciences, Tabriz University of Medical Sciences, Tabriz, Iran

**Keywords:** Taurine, Diabetes, Oxidative stress, Inflammation

## Abstract

**Background:**

Reduced serum level of taurine in type 2 diabetes mellitus (T2DM) was shown to be associated with the metabolic alterations and clinical complications of diabetes. Dietary supplementation with taurine may attenuate oxidative stress and inflammatory responses in T2DM as well as alleviate diabetes-induced complications. Hence, this study evaluated the effect of taurine supplementation on oxidative stress and inflammatory biomarkers in patients with T2DM.

**Methods:**

Fifty patients with T2DM were randomly allocated to two groups to consume either taurine (containing 1000 mg taurine), or placebo (containing crystalline microcellulose) three times per day for 8 weeks. Anthropometric data, dietary intake, serum total antioxidant capacity (TAC), malondialdehyde (MDA), the activities of antioxidant enzymes superoxide dismutase (SOD) and catalase (CAT), serum levels of tumor necrosis factor alpha (TNF-α), interleukin 6 (IL-6) and high-sensitivity C-reactive protein (hs-CRP) were assessed before and after intervention.

**Results:**

There was a significant increase in SOD (5.1%, *p *= 0.004) and CAT (4.22%, *p *= 0.001) after 8 weeks of taurine supplementation. In addition, serum levels of MDA (26.33%, *p *= 0.001), hs-CRP (16.01%, *p *= 0.001), and TNF‐α (11.65%, *p *= 0.03) significantly decreased in the taurine group compared with baseline. Following treatment, the taurine group had fewer serum levels of MDA (*p *= 0.04), hs-CRP (*p *= 0.002) and TNF-α (*p *= 0.006) than the placebo group. Also, a significant increase was observed in SOD (*p *= 0.007), and CAT (*p *= 0.001) in the taurine group compared with the placebo group. There were no differences in the serum levels of IL-6 or TAC.

**Conclusions:**

The findings of this study showed that taurine supplementation improved some oxidative stress indices and inflammatory biomarkers in patients with T2DM.

*Trial registration* The protocol of this clinical trial is registered with the Iranian Registry of Clinical Trials (http://www.IRCT.IR, identifier: IRCT20121028011288N16).

## Background

Type 2 diabetes mellitus (T2DM), as the most prevalent endocrine disease in the world, is characterized by chronic hyperglycemia, impaired pancreatic beta cell function, and insulin resistance in the target organs [1]. In particular, longer diabetes duration increases the risk of DM complications in diabetic patients diagnosed at a younger age [2]. Comorbid conditions, including hypertension and dyslipidemia, increase DM complications and thereby add to its overall morbidity and mortality [3, 4]. In particular, diabetic dyslipidemia, which is characterized by decreased levels of high-density lipoprotein (HDL-C), increased levels of very low-density lipoprotein (VLDL), total cholesterol, and synthesis of atherogenic low-density lipoprotein (LDL) particles, is associated with atherosclerotic lesions [4, 5]. However, the exact mechanisms underlying diabetes dyslipidemia are not completely understood, although visceral obesity, and subsequent insulin resistance partially explain impaired lipid metabolism [6]. Chronic hyperglycemia, along with insulin resistance following T2DM, is associated with increased oxidative stress and inflammation in various organs, which can lead to macrovascular and microvascular complications [7]. On the other hand, increased levels of reactive oxygen species (ROS) and pro-inflammatory agents in target tissues are the most important determinants in patients susceptible to developing insulin resistance [8]. Therefore, mitigating oxidative stress and inflammation are major targets for prevention, management, and treatment of T2DM [9]. This has led researchers to investigate new compounds with strong antioxidant and anti-inflammatory activities.

Taurine is a sulfuric amino acid of the chemical formula 2-aminoethanesulfonic acid NH2CH2CH2SO3H that is abundant in mammalian tissues [10]. This semi-essential amino acid is provided in the body both from food intake and from the reaction of methionine with cysteine in the liver [11]. Evidence suggests that taurine may be a conditionally essential amino acid in diseases associated with increased oxidative stress and inflammation, such as diabetes mellitus, obesity, metabolic syndrome, and atherosclerosis [9, 12]. Several studies have found an inverse association between plasma taurine concentrations and fasting plasma sugar (FBS) as well as diabetes complications, suggesting taurine has a protective role in the progression of diabetes [13, 14]. As a functional nutrient, taurine is involved in osmoregulation, detoxification, calcium homeostasis, neuromodulation, and cytoprotection [10, 15, 16]. Various studies have found regulatory impacts of taurine on the production of proinflammatory cytokines, oxidative stress, lipid profile, and blood pressure [15]. In addition, taurine appears to interfere with the insulin signaling pathway and to affect β-cell insulin secretion, leading to better control of glucose metabolism [17].

Although the modulatory effects of taurine supplementation on oxidative stress and inflammatory biomarkers have been frequently reported in animal models of diabetes [18–21], there are limited clinical trials. To date, no human data are available on the anti-inflammatory effects of taurine in patients with T2DM, and there has been only one trial on its antioxidative effects [22]. Therefore, this clinical trial explored the effects of taurine supplementation on oxidative stress indices and inflammation in patients with T2DM.

## Materials and methods

### Study design

This randomized parallel clinical trial was conducted at the Diabetes Association and Endocrine Clinic, Imam Reza Hospital, Tabriz University of Medical Sciences, from February 8, 2019 to May 20, 2019. According to the sample size formula for randomized clinical trials [$n = \frac{{2 Sp^{2} \left( {Z_{{1 - \frac{\alpha }{2}}} + Z_{1 - \beta } } \right)^{2} }}{{(\mu 1 + \mu 2)^{2} }}$], considering an α of 0.05, a β of 0.20 (for a power of 80%), and blood concentration of insulin as a key outcome, the sample size was calculated at 19 per group [23]. A total of 185 potentially suitable volunteers referred to the clinic of Imam Reza Hospital were screened for eligibility by a dietitian. Finally, 50 eligible subjects were enrolled in the study, 26 in the taurine group and 24 in the placebo group.

Prior to beginning the study, written informed consent was obtained from all participants. Individuals diagnosed with T2DM with age between 30 and 60 years and a body mass index (BMI) between 25 and 35 kg/m^2^ were eligible for participation. Exclusion criteria were the use of insulin, being treated with non-steroidal anti-inflammatory drugs or lipid-lowering drugs, intake of supplements within the past 3 months, and changes in physical activity and dietary intake during the study. Patients diagnosed with polycystic ovary syndrome, chronic renal and liver disease, cardiovascular diseases, hypothyroidism, hyperthyroidism, malignancies, gastrointestinal dysfunction and infectious diseases, or patients with physiological conditions such as pregnancy or lactation were also excluded. After stratification for sex, age and BMI values, participants were randomly assigned to consume either taurine or placebo for 8 weeks. Randomization was carried using a block randomization process with a computer-generated random-number table by a statistician. Subjects in the taurine group received capsules containing 1000 mg taurine (Karen Pharma and Food Supplement Co., Iran, > 99% pure) three times a day for 8 weeks following each meal and those in the placebo group received capsules containing microcrystalline cellulose three times daily for the same duration. The appearance of the placebo capsules, such as shape, color and size, was identical to the taurine capsules. The participants were requested to maintain their usual physical activity or dietary intake throughout the experimental period. In order to evaluate the acceptance of the intervention by the participants, they were asked to return the bottle. Participants with good compliance (defined as consumption of over 90% of the capsules) were included in the analysis. Participants and researchers were blinded to the treatment allocation. The study was performed according to the principles provided in the Declaration of Helsinki. The research protocol received the approval of the Ethical Committee of the Tabriz University of Medical Sciences, and was registered with the Iranian Registry of Clinical Trials (http://www.IRCT.IR, identifier: IRCT20121028011288N16). The reporting of this manuscript adheres to CONSORT guidelines [24].

### Measurements of anthropometric indices, food intake and physical activity

After completing a general questionnaire on demographic information, medical history, and medication use [25], height and body weight were measured using a wall mounted stadiometer and a Seca scale (Seca, Germany), without shoes and wearing light clothing, respectively. Body mass index was calculated as weight (kg) divided by height^2^ (m). Waist circumference was measured midway between the lowest rib and the iliac crest and hip circumference was measured around the widest portion of the hip using a non-elastic tape. Data on dietary intake was collected using a 24-h recall procedure across 3 days: two non-consecutive normal days and one holiday. Three-day mean values of energy, macronutrient and micronutrient intakes of all participants were calculated using Nutritionist 4 software (First Databank Inc., Hearst Corp., San Bruno, CA, USA). To evaluate physical activity, all participants completed the International Physical Activity Questionnaire (IPAQ) at baseline and following the eight weeks of the intervention [26].

### Blood sampling and biochemical assays

At baseline and after 8 weeks of intervention, 8 mL fasting venous blood samples were collected from each participant. The blood sample was centrifuged at 2500 rpm for 10 min (Orum Tadjhiz Centrifuge, Iran) to separate serum samples, which were kept frozen at − 70 °C until use. Serum total antioxidant capacity (TAC) and malondialdehyde (MDA) were determined using standard spectrophotometry methods and a commercially available total antioxidant capacity assay kit (Navand Salamat, Iran). Reliable spectroscopy methods with commercial kits (RANDOX Lab, Crumlin, UK) were used to assess the activity of the antioxidant enzymes superoxide dismutase (SOD) and catalase (CAT) in red blood cells. Serum glucose was determined by the standard enzymatic method with a Pars Test Kit (Karaj, Iran). Serum insulin concentration was obtained by an ELISA assay with a Monobind kit (USA). Serum tumor necrosis factor alpha (TNF‐α) and Interleukin 6 (IL-6) were determined using ELISA (Human ELISA kit, Crystal Day Bio-Tec). Finally, the serum level of hs-CRP was determined using the immunoturbidimetric method (Bio System Kit, Spain).

### Statistical analysis

All data were analyzed using SPSS 23 software (IBM/SPSS Inc., Chicago, IL, USA). Data analysis were carried out based on intention‐to‐treat (ITT) method. The ITT population included all enrolled and randomized participants. The multiple imputation method was used to impute missing values. The Kolmogorov–Smirnov test was used to determine the distribution of data. Quantitative variables with normal distribution were expressed as mean ± standard deviation. Median and interquartile range (IQR) were used to present variables with skewed distribution. A Chi square test was conducted for comparison of qualitative variables. Independent sample Student’s *t* test and Mann–Whitey test, respectively, were used to compare the percent change of the quantitative parametric data and variables with non-parametric distribution between the intervention and placebo groups. Log transformation was performed for variables with skewed distribution before they were included in the analysis. To detect within-group changes, paired-sample t-tests were used for quantitative parametric data, while data with skewed distribution were analyzed using Wilcoxon’s rank-sum test. Analysis of covariance (ANCOVA) was used to compare mean values of study variables between groups adjusted for differences in baseline values, weight, and energy intake. Statistical significance was considered to be *p* < 0.05 at a 95% confidence interval (95% CI).

## Results

**Fig. 1:**
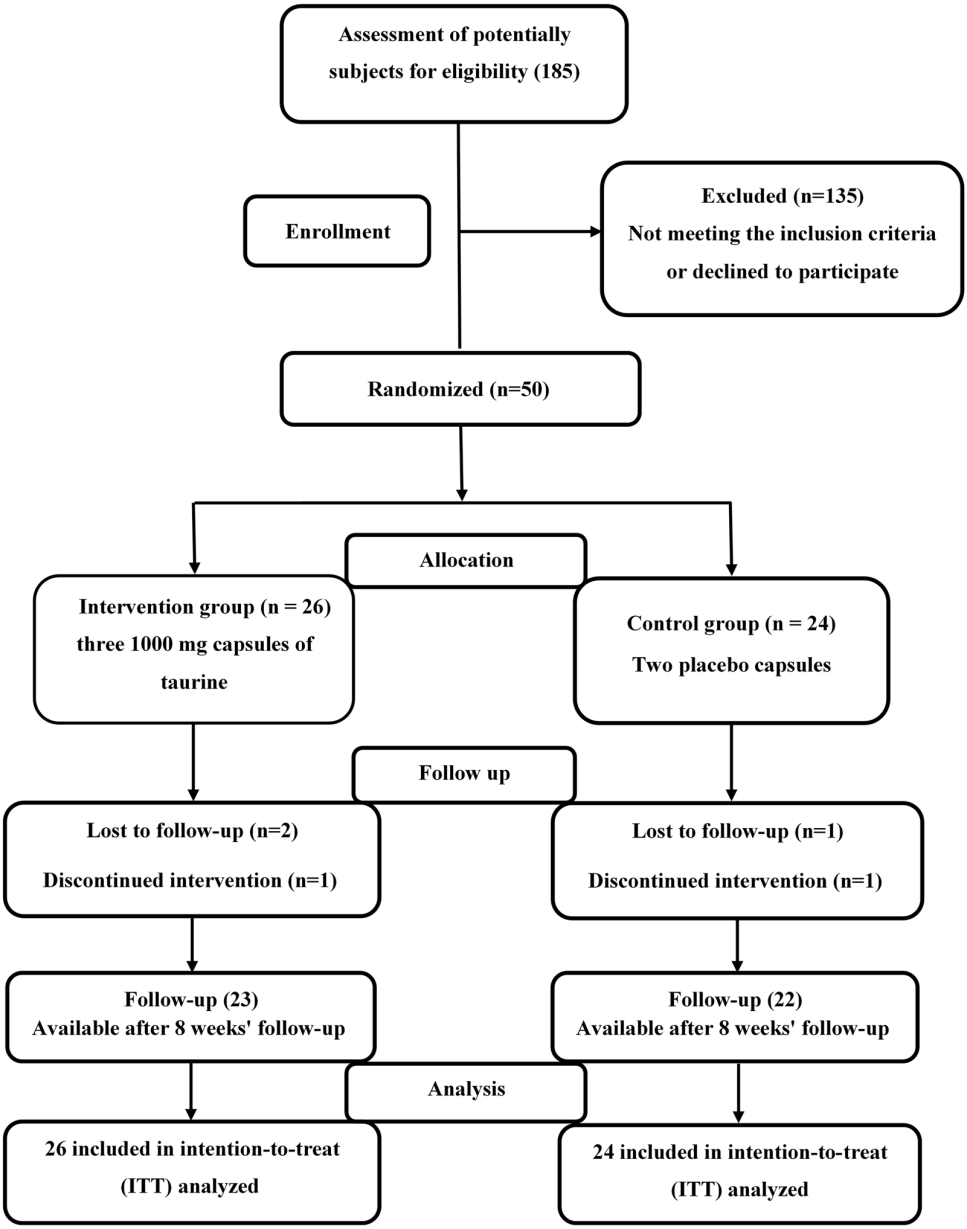
Flow diagram of patient recruitment and randomization process

The patient recruitment and randomization process is summarized in a flow diagram in Fig. 1. The 50 subjects were assigned to either taurine (n = 26) or placebo (n = 24) groups. Forty-five patients completed the study (23 patients in the taurine group and 22 in the placebo group). Three patients in the taurine group were excluded because of physical illness (n = 2) or insulin therapy (n = 1). Two subjects in the placebo group were also excluded for the following reasons: travel (n = 1) or hospitalization (n = 1). Finally, 50 patients were analyzed by the intention‐to‐treat (ITT) method.

**Fig. 2:**
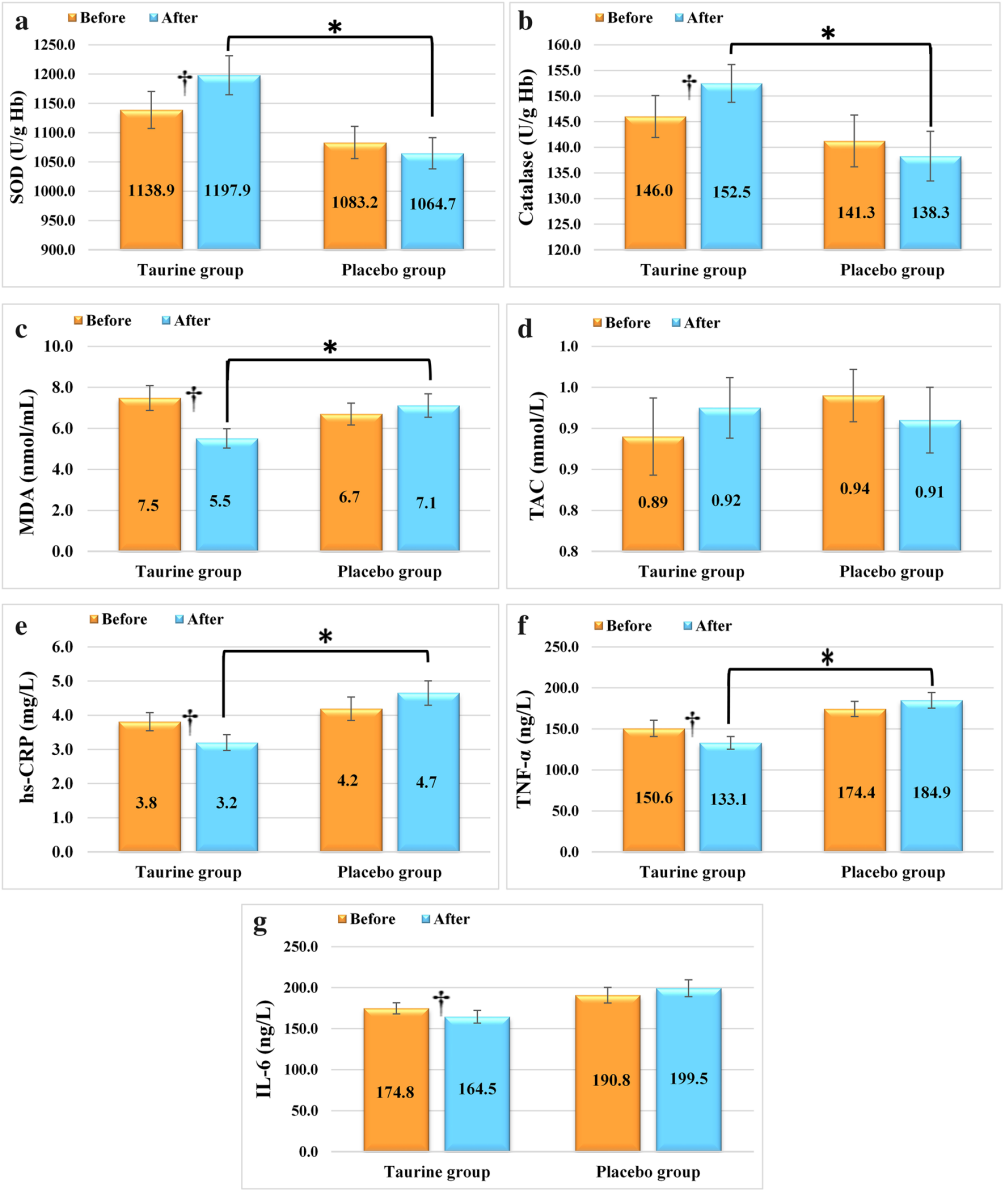
Effects taurine supplementation on **a** superoxide dismutase (SOD) [reference value: 100–1600 μ/g Hb] **b** catalase (CAT) [reference value: 1.12–150 μ/mL] **c** malondialdehyde (MDA) [reference value: 0.38–133.33 nmol/mL], **d** total antioxidant capacity (TAC) [reference value: 0.049–2.5 mmol/L], **e** high-sensitivity C-reactive protein (hs-CRP) reference value: 0.10–500 mg/L], **f** tumor necrosis factor alpha (TNF‐α) [reference value: 3–900 ng/L] and **g** serum interleukin 6 (IL-6) [reference value: 2–600 ng/L]. Data were present as mean ± SE for taurine (n = 26) and placebo (n = 24) groups. **†**P < 0.05 for within group comparisons (paired sample t test); *P < 0.05 for between group comparisons (ANCOVA adjusted for differences in baseline values, weight, and energy intake)

Baseline characteristics of participants are shown in Table 1. There were no significant differences in mean age in years (taurine group: 42.04 ± 6.37; placebo group: 44.17 ± 658), duration of disease in years (taurine group: 3.85 ± 1.58; and placebo group: 3.25 ± 1.30), sex ratio, family history of diabetes, number and  % of patients using glucose-lowering agents/drugs, blood glucose levels, HbA1C,  %, blood insulin levels, weight, BMI, WC, or physical activity between groups at baseline. No statistical differences were found in energy, macronutrient, or micronutrient intakes within or between the two groups at baseline or after the trial completion as well as after adjusting for confounders. The changes in oxidative stress and circulating inflammatory biomarkers are shown in Fig. 2. At the start of the clinical trial, there were no significant differences between the taurine group and the placebo group for superoxide dismutase (SOD), catalase (CAT), malondialdehyde (MDA), total antioxidant capacity (TAC), high-sensitivity C-reactive protein (hs-CRP), Tumor Necrosis Factor-α (TNF-α), or interleukin 6 (IL-6) concentrations (*p* > 0.05).

**Table 1:** General characteristics of T2DM patients at baseline

Variable	Taurine group (n = 26)	Placebo group (n = 24)	P-value
Age (years)^b^	42.04 ± 6.37	44.17 ± 658	0.252^d^
Sex (n/%)			0.630^c^
Male (n/%)^a^	7 (26.9)	7 (33.3)	
Female (n/%)^a^	19 (73.1)	16 (66.7)	
Duration of T2DM, years^b^	3.85 ± 1.58	3.25 ± 1.30	0.212^d^
Family history of diabetes (%)^a^	11 (42.3)	9 (37.5)	0.828^d^
Metformin use, n (%)^a^	24 (92.3)	22 (91.7)	0.935^c^
Glibenclamide use, n (%)^a^	8 (30.8)	6 (25)	0.658^c^
Weight (kg)^b^	80.21 ± 8.49	78.90 ± 11.7	0.657^d^
BMI (kg/m^2^)^b^	30.62 ± 3.98	30.25 ± 2.66	0.699^d^
WC (cm)^b^	100.53 ± 7.01	101.25 ± 6.28	0.708^d^
FBS (mg/dL)^b^	168.37 (16.33)	174.22 (19.79)	0.259^d^
HbA1C, %^b^	6.97 (0.71)	7.26 (0.91)	0.215^d^
Insulin, mU/mL^b^	10.66 (3.17)	11.70 (3.60)	0.340^d^
Physical activity			
Low (%)^a^	11 (42.3)	14 (58.3)	0.511
Moderate (%)^a^	14 (53.8)	8 (33.3)	
High (%)^a^	1 (4.3)	2 (8.3)	

T2DM: Type 2 Diabetes Mellitus; BMI: Body Mass Index; WC: Waist Circumference; FBS: Fasting Blood Sugar

^a^Values are expressed as frequency (%)

^b^Values are expressed as mean (SD)

^c^Chi square test

^d^Independent samples t-test

In the placebo group, TAC, SOD and CAT all decreased after 8 weeks; however, in the taurine group, compared to baseline values, a significant increase of SOD 5.1%, p = 0.004, was recorded in SOD compared to a decrease of 1.7% in the placebo group*, p *= 0.283; a significant increase of 4.22%, *p *= 0.001, in CAT, compared to a decrease of 2.08% in the placebo group, *p *= 0.154, whereas there was no significant difference in TAC (*p *= 0.133) compared to baseline values. A significant decrease in MDA of 26.33%, *p *= 0.001, was seen in the taurine group, compared to an increase of 6.11% in the placebo group, *p *= 0.649. Covariance analysis (ANCOVA) revealed a significant increase in SOD (*p *= 0.007) and CAT (*p *= 0.001), as well as a significant reduction in MDA (*p *= 0.04) in the taurine group compared with the placebo group after adjusting for baseline variables, duration of T2DM, weight, and calorie intake changes; however, no significant difference was observed in TAC (*p *= 0.06).

To determine the effect of taurine on inflammation, the serum levels of hs-CRP, TNF-α, and IL-6 were compared at baseline and after 8 weeks of the intervention. Intake of taurine supplements led to a significant decrease in hs-CRP of 16.01%, *p *= 0.001, compared to an increase of 10.73% in the placebo group, *p *= 0.153; a decrease of 11.65%in TNF-α, *p *= 0.03, compared to an increase of 6.06% in the placebo group, *p *= 0.188; and a decrease in IL-6 of 5.89%, *p *= 0.04, compared to an increase of 4.5% in the placebo group, *p *= 0.372 after 8 weeks in comparison to baseline values. ANCOVA of changes between groups showed a significant decrease in hs-CRP (*p *= 0.002) and TNF-α (*p *= 0.006) in the taurine group compared to the placebo group, following adjustments for baseline values, duration of T2DM, weight, and calorie intake, whereas the changes in IL-6 (*p *= 0.07) were not significant. No adverse effects were reported during the study period.

## Discussion

To the best of our knowledge, this is the first report of a controlled clinical trial in individuals with T2DM to examine the effects of taurine supplementation on oxidative stress indices and inflammatory biomarkers. The results of this study showed some significant improvements in oxidative stress parameters, which suggests that taurine has an antioxidative component. In this study, taurine supplementation caused a significant reduction in serum levels of MDA as well as an increase in the activities of the antioxidant enzymes SOD and CAT. Also, a positive effect was seen in the case of TAC in the taurine group, albeit without reaching significance after adjusting for covariates.

Our results correspond to those found in experimental studies, such as the study by Shivananjappa et al. in which MDA and ROS content decreased and activities of antioxidant enzymes SOD and CAT increased after oral supplementation of pregnant DM rats with taurine [27]. Wong et al. also found an improvement in SOD and MDA in rats with DM after administration of taurine [28], and Rashid et al. reported significant increases in SOD and CAT as well as a significant decrease in ROS production after administration of taurine in rats with DM [29].

Relatively few clinical trials have explored changes in oxidative stress following consumption of taurine and the results of these trials are contradictory. In a clinical trial in which obese women received 3000 mg/d taurine for 8 weeks, a significant reduction occurred in lipid peroxidation (20%), though advanced oxidation protein products, ferric-reducing antioxidant power, and GSH levels did not show significant changes [23]. A trial of 11 healthy young men who received either a taurine supplement (2 grams three times a day) or placebo for 7 days prior to a second exercise test [30] found a significant decrease in exercise-induced oxidative stress as well as a significant reduction in DNA migration. In a trial among T2DM patients, supplementation with 3000 mg/d of taurine for 4 months did not affect oxidative stress status [31]; however, this study had a small sample size due to its focus on newly diagnosed T2DM patients along with poor glycemic control, which might explain the lack of significant changes in oxidative stress indices.

Although the mechanisms underlying the antioxidant effects of taurine are not well understood, scavenging ROS, interfering with ROS activity, and regeneration of thiol groups may be the most likely mechanisms [12]. In particular, studies suggest that taurine suppresses the production of superoxide in the mitochondria [32]. Overall, the stimulatory effect of taurine on SOD [28], CAT [27, 29] and GPx [27] enzyme activity results in a marked reduction in ROS generation. In addition, taurine, as an end product of cysteine metabolism, contributes to the maintenance of GSH levels [33]. The glucose-lowering effect of taurine may suppress advanced glycation end products generation due to prolonged exposure to hyperglycemia [34].

Our findings on the effects of taurine supplementation on the inflammatory response were noteworthy. Chronic low-grade inflammation following chronic hyperglycemia in T2DM leads to impaired pancreatic beta cell function and exacerbated insulin resistance [35]. In this study, we observed decreased inflammatory hs-CRP and TNF-α concentrations without significant changes in IL-6 after taurine intervention.

These results agree with animal models that analyzed the effects of taurine on the regulation of inflammatory mediators. In line with these results, taurine supplementation significantly decreased TNF-α in mice who were fed a high-fat diet [36]. Supplementation of DM rats with a lower dose of taurine for a shorter term compared to the previous study showed a significant decrease in NF-κB gene expression and a decrease in TNF-α and IL-6 levels [37]. The same findings were observed in a study by Pei et al. in which taurine supplementation suppressed gene expression of TNF-α and deleterious effects of arsenic on beta-pancreatic cells [38], as well as a study by Abd El-Twab et al., in which taurine significantly decreased TNF-α and IL-6 in rats with DM [19]. A clinical trial found that the supplementation of obese women with 3000 mg/day of taurine for 8 weeks improved hs-CRP levels by 29%; however, changes in TNF-α and IL-6 were not significant [23]. It appears that the attenuation of oxidative stress resulting from neutralization of hypochlorous acid by forming taurine chloramine mediates the anti-inflammatory impact of taurine [39]. Taurine chloramine was also shown to suppress the production of inflammatory cytokines such as hs-CRP, TNF-α and IL-6 by inhibiting NF-κB activity as a key mediator of inflammation [32, 40].

Although this study was the first randomized, double-blind, placebo-controlled trial to assess the effect of taurine on inflammatory biomarkers in patients with T2DM, this study has several limitations. These include a short intervention period and a small sample size which are suggested to be considered for future studies. In addition, other oxidative stress indices (such as glutathione peroxidase or thiobarbituric acid reactive substances) and inflammatory biomarkers (including interleukin 1 (IL-1) and monocyte chemoattractant protein-1) are candidates for evaluation following taurine supplementation in patients with T2DM. Given the close link between taurine deficiency and metabolic impairment along with a high prevalence of taurine deficiency in DM, measuring plasma levels of taurine is highly recommended. Hence, the lack of measurement of plasma levels of taurine due to the lack of laboratory capacity is considered a third limitation.

## Conclusion

As a whole, this study found that taurine supplementation in patients with T2DM improved oxidative stress indices and reduced levels of inflammatory biomarkers. However, while these results are generalizable to T2DM, it remains to be seen whether these results can be generalized to smokers, or to patients with T1DM. In future research, larger sample sizes and a longer intervention period may be indicated to confirm the efficacy of taurine in affecting clinical differences and preventing complications of T2DM.

## Data Availability

Please contact authors for data request.

## Abbreviations

ALX: alloxan
BMI: body mass index
CAT: catalase
FBS: fasting blood sugar
GPx: glutathione peroxidase
hs-CRP: high-sensitivity C-reactive protein
IDA: International Diabetes Association
IL-1: interleukin 1
IL-6: interleukin 6
IPAQ: International Physical Activity Questionnaire
MDA: malondialdehyde
NF- κB: nuclear factor kappa beta
ROS: reactive oxygen species
SOD: superoxide dismutase
STZ: streptozotocin
T2DM: type 2 diabetes
TAC: total antioxidant capacity
TNF-α: tumor necrosis factor alpha
WC: waist circumference
